# Spontaneous Uterine Rupture in a Preterm Pregnancy following Myomectomy

**DOI:** 10.1155/2016/6195621

**Published:** 2016-01-26

**Authors:** Claire Sutton, Prue Standen, Jade Acton, Christopher Griffin

**Affiliations:** ^1^Department of Obstetrics and Gynaecology, King Edward Memorial Hospital, 374 Bagot Road, Subiaco, WA 6008, Australia; ^2^Department of Maternal Fetal Medicine, King Edward Memorial Hospital, 374 Bagot Road, Subiaco, WA 6008, Australia

## Abstract

A 44-year-old nulliparous woman was transferred to a tertiary obstetric hospital for investigation of acute onset abdominal pain. She was at gestation of 32 weeks and 2 days with a history of previous laparoscopic fundal myomectomy. An initial bedside ultrasound demonstrated oligohydramnios. Following an episode of increased pain early the following morning, a formal ultrasound diagnosed a uterine rupture with the fetal arm extending through a uterine rent. An uncomplicated classical caesarean section was performed and the neonate was delivered in good condition but with a bruised and oedematous right arm. The neonate was transferred to the Special Care Nursery for neonatal care. The patient had an uncomplicated postoperative course and was discharged home three days following delivery. This is an unusual presentation of uterine rupture following myomectomy where the fetal arm had protruded through the uterine wall.

## 1. Introduction

Uterine rupture is an obstetric emergency that can result in significant maternal and fetal morbidity and mortality. It is defined as a full-thickness separation of the uterine wall and overlying visceral peritoneum [1]. The most important contributing risk factor is the presence of a scarred uterus, usually secondary to uterine surgery such as a myomectomy or caesarean section. We report a case of spontaneous antenatal uterine rupture at gestation of 32 weeks and 2 days, originating from a myomectomy scar. The fetal arm was seen on ultrasound to be protruding out through the uterine rupture on the background of a reactive fetal cardiotocogram (CTG).

## 2. Case Presentation

A 44-year-old nulliparous woman was transferred to an Australian tertiary maternity centre with severe acute onset abdominal pain at gestation of 32 weeks and 2 days. Maternal observations on admission were within normal limits and a fetal CTG demonstrated a reactive trace. Following admission, the pain intensified and a bedside ultrasound demonstrated oligohydramnios and a fetus in breech presentation. The patient was commenced on oral antibiotics and received intramuscular corticosteroids for presumed premature prolonged prelabour rupture of membranes.

The patients past medical history included a laparoscopic fundal myomectomy performed eight years ago at a private health care facility. In addition to this her medical history included a laparoscopic ovarian dermoid cystectomy, gastric band insertion, and a provoked deep vein thrombosis (DVT), for which the patient no longer required anticoagulation.

**Figure 1:**
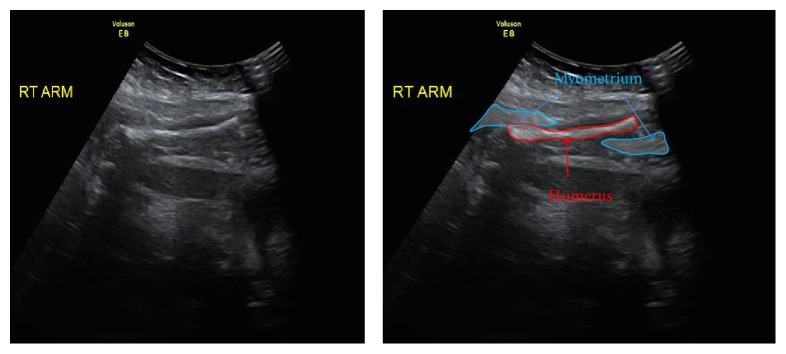
Fetal ultrasound showing uterine rupture and intra-abdominal right fetal arm.

The pain intensified during the night and a formal ultrasound was performed early the next morning. This ultrasound confirmed anhydramnios and uterine rupture with the right fetal arm seen intra-abdominally (Figure 1). Fetal growth parameters were within normal limits with the estimated fetal weight plotted on the 50th centile.

**Figure 2:**
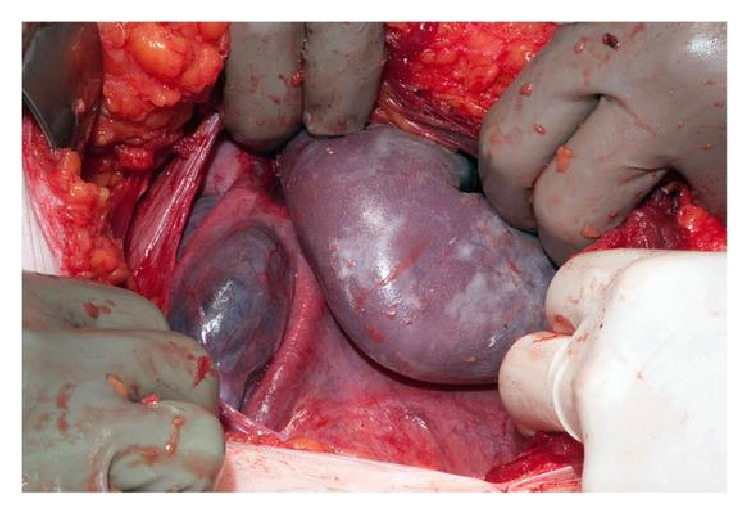
Fetal arm protruding through the uterus at laparotomy to the level of the fetal shoulder.

The patient was transferred to theatre for an urgent caesarean section at gestation of 32 weeks and 3 days. A midline laparotomy was performed and the right fetal arm was seen protruding through a ruptured anterior myomectomy scar to the level of the fetal shoulder (Figure 2).

**Figure 3:**
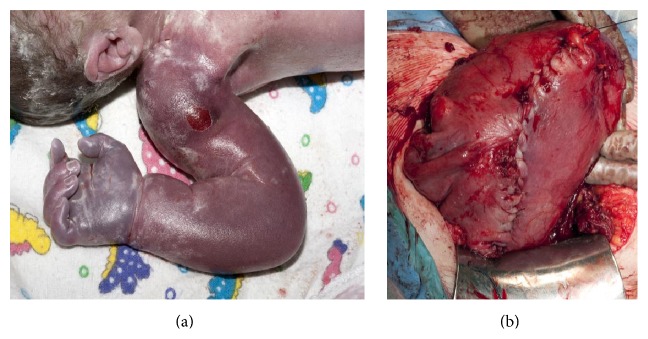
(a) Oedematous right fetal arm immediately following delivery. (b) Double layer closure of the extended uterine incision.

The fetal right arm and hand were oedematous and bruised but otherwise uncompromised (Figure 3(a)). The neonate was delivered via a classical caesarean section, which extended the uterine rupture into an inverted “J” shape and allowed for delivery without additional trauma to the neonate or mother. This classical incision was then closed in two layers with mass closure of the abdominal wall (Figure 3(b)). As the site of the rupture was at the avascular uterine scar, there was minimal blood loss from the uterus. Presumably, the fetal arm also provided a degree of compression and haemostasis.

The patient was advised against future pregnancies due to the increased risk of uterine rupture in subsequent pregnancies. She went on to have an uncomplicated postoperative course and was discharged home three days following delivery. The neonate was transferred directly to the Special Care Nursery and had an uncomplicated neonatal course. A Doppler ultrasound of the neonate's right arm, performed on day one, did not show any arterial stenosis or venous thrombosis.

## 3. Discussion

The increasing incidence of recognised uterine rupture is well documented [2]. The incidence varies widely between the developed and developing world, as do the antecedent risk factors and clinical outcomes. From 1976 to 2012, 25 peer-reviewed publications described an overall uterine rupture rate of 1 in 1,146 pregnancies or 0.07% [2]. In the developed world, virtually all uterine ruptures occur in the setting of a scarred uterus [3]. The rate of uterine rupture in an unscarred uterus is 0.006% [4]. This increases to 0.5% following one caesarean section and to 2% with two or more previous caesarean sections [2].

The incidence of uterine rupture following myomectomy is less well documented and evidence in this area remains limited due to its rarity. Uterine rupture after abdominal myomectomy (AM) is uncommon with the incidence reported in the literature ranging from 0.24 to 5.3% [5]. The increasing number of case reports describing uterine rupture following laparoscopic myomectomy (LM) has, however, raised the question of whether the risk of uterine rupture is greater following LM compared with AM.

Dubuisson et al. performed an observational study of 145 pregnancies following LM, with 100 live deliveries and one case of uterine rupture from the myomectomy scar (incidence of 1%) [6]. More recently, a larger study by Koo et al. of 523 women who became pregnant following LM again reported that major complications in this population are rare [7]. Importantly, they reported a uterine rupture incidence of 0.6%, all of which occurred in nulliparous females who had never laboured [7]. 76.5% of the women included in this study reached term gestation, and 19.1% laboured successfully [7].

Current literature suggests that the risk of uterine rupture following LM may depend on the characteristics of the myomectomy performed and surgical techniques used. The size and number of myomas removed whether the endometrial cavity has been entered or not and the surgical techniques employed to achieve haemostasis and uterine closure have been identified as potential factors affecting the risk of uterine rupture in subsequent pregnancies. However, due to limited evidence in this field these factors remain a topic for debate.

Bernardi et al. in 2014 reviewed 55 pregnancies that followed LM and found a uterine rupture rate of 10% within a follow-up period of 73.55 months [5]. Uterine rupture in these cases was found to occur in patients with a short (<12 months) LM to conception interval, cases where the endometrial cavity had been entered at myomectomy and those in which large (diameter > 4 cm) fibroids had been removed [5]. Expert opinion (level III) recommends that intraoperative strategies to reduce uterine rupture in subsequent pregnancies include multilayer uterine closure, avoidance of entry into the endometrial cavity, avoidance of excessive electrosurgery to reduce devascularization, and prevention of haematoma formation, which may affect wound strength [8, 9].

Uterine rupture most commonly presents intrapartum and is a clinical diagnosis based on alterations in the fetal heart rate pattern, maternal signs of shock, vaginal bleeding, and/or abdominal pain [3]. These signs and symptoms are usually identified in hospitalised patients undergoing monitoring before or during labour. In patients who experience uterine rupture in an out-of-hospital setting and then present to a health care facility, most arrive in a state of shock requiring urgent resuscitation and surgery [10, 11]. The management of suspected uterine rupture should include a prompt diagnosis followed by timely and definitive surgical management with concurrent maternal haemodynamic stabilisation [2]. The recommended time for successful intervention after uterine rupture but before the onset of major fetal morbidity is between 10 and 37 minutes [2]. Definitive surgical treatment options include scar repair or hysterectomy.

The consequences of uterine rupture affect both the mother and the fetus. Maternal consequences of uterine rupture include hypovolaemic shock secondary to haemorrhage, genitourinary injury, potential need for hysterectomy, and maternal death [2]. The fetal consequences include fetal hypoxia, fetal acidosis, and fetal or neonatal death [2]. In 2003, Chauhan et al. estimated that the overall rate of hysterectomy secondary to uterine rupture is 0.09% with a perinatal mortality rate of 0.04% and a maternal mortality rate of 0.02% [12].

There is currently insufficient evidence to determine the recommended mode of delivery following myomectomy. Options include an elective lower uterine segment caesarean section (ELUSCS) and a trial of labour, with some women being offered an ELUSCS by clinicians with the view that it reduces the risk of uterine rupture [8]. In addition to the correct mode of delivery, this case report raises the debate of optimal timing for delivery, particularly given that rupture occurred at a preterm gestation of 32 weeks and 3 days. Kiseli et al. report a similar case of a uterine rupture at a preterm gestation of 23 weeks occurring one year after a laparoscopic fundal myomectomy [13]. This case presented in a similar way with acute onset diffuse abdominal pain and was managed with laparotomy and repair of uterine rupture at the site of previous myomectomy with three-layer closure [13]. The timing of uterine rupture, therefore, appears to be unpredictable and is unlikely to provide guidance with regard to the optimal timing for delivery.

## 4. Conclusion

Uterine rupture during pregnancy is a rare event; however, the incidence is increasing. This patient's previous laparoscopic myomectomy was the greatest risk factor for rupture. The key steps for successful management of uterine rupture include prompt diagnosis followed by definitive surgical management with concurrent maternal haemodynamic stabilisation [2].
